# Creating Prognostic Systems for Well-Differentiated Thyroid Cancer Using Machine Learning

**DOI:** 10.3389/fendo.2019.00288

**Published:** 2019-05-08

**Authors:** Charles Q. Yang, Lauren Gardiner, Huan Wang, Matthew T. Hueman, Dechang Chen

**Affiliations:** ^1^Department of Otolaryngology, Walter Reed National Military Medical Center, Bethesda, MD, United States; ^2^Class of 2020, Virginia Commonwealth University School of Medicine, Richmond, VA, United States; ^3^Department of Biostatistics, The George Washington University, Washington, DC, United States; ^4^Department of Surgical Oncology, John P. Murtha Cancer Center, Walter Reed National Military Medical Center, Bethesda, MD, United States; ^5^Department of Preventive Medicine & Biostatistics, F. Edward Hébert School of Medicine, Uniformed Services University of the Health Sciences, Bethesda, MD, United States

**Keywords:** thyroid cancer, cancer staging, C-index, dendrogram, machine learning, survival

## Abstract

Updates to staging models are needed to reflect a greater understanding of tumor behavior and clinical outcomes for well-differentiated thyroid carcinomas. We used a machine learning algorithm and disease-specific survival data of differentiated thyroid carcinoma from the Surveillance, Epidemiology, and End Results Program of the National Cancer Institute to integrate clinical factors to improve prognostic accuracy. The concordance statistic (C-index) was used to cut dendrograms resulting from the learning process to generate prognostic groups. We created one computational prognostic model (7 prognostic groups with C-index = 0.8583) based on tumor size (T), regional lymph nodes (N), status of distant metastasis (M), and age to mirror the contemporary American Joint Committee on Cancer (AJCC) staging system (C-index = 0.8387). We showed that adding histologic type (papillary and follicular) improved the survival prediction of the model. We also showed that 55 is the best cutoff of age in the model, consistent with the changes from the most recent 8th edition staging manual from AJCC. The demonstrated approach has the potential to create prognostic systems permitting data driven and real time analysis that can aid decision-making in patient management and prognostication.

## Introduction

The American Joint Committee on Cancer (AJCC) has released eight iterations of its Cancer Staging Manual since initial publication in 1976. The tumor, nodal involvement, metastasis (TNM) staging model it publishes is a mechanism for comparing like or unlike groups of cases. This is the worldwide standard for the way cancer information is communicated and is used for staging and prognosis, treatment recommendations, and research at all levels. Periodic improvements attempt to integrate new factors that inform behavior and prognosis of each cancer.

The accumulation of survival data with current advances in cancer research can improve the delivery of patient care following enhanced prognostic accuracy of staging cancers. The 8th edition of AJCC asserted major changes in the staging of oropharyngeal and breast cancers due to the increasing wealth of clinical data. Hormone receptor status and histologic grade were incorporated in the traditional TNM staging of breast cancer, whereas HPV status received its own staging algorithm in oropharyngeal cancers (1). Updates to our staging models reflect a greater understanding of tumor behavior and clinical outcomes, and further refinement of the staging model for well-differentiated thyroid carcinomas is needed.

Well-differentiated thyroid carcinomas generally demonstrate different clinical behavior compared to medullary and anaplastic thyroid carcinomas. Current clinical outcomes of well-differentiated thyroid cancers, which include papillary and follicular carcinoma, are largely guided by the age of the patient. Historically, the age cutoff for staging was 45 years as it is the mean dataset age upon which staging recommendations are based (2). Changing the age cutoff in prognostic staging from 45 to 55 has led to the down staging of 12% of patients whose mortality would have otherwise been overstated. The age cutoff of 55 could improve the prognostic validity of the AJCC staging model (2).

In addition to AJCC's widely used TNM system, numerous prognostic calculators have been described to inform prognosis for new cases of differentiated thyroid cancer. The European Organization for Research on Treatment of Cancer (EORTC) is a historical system from 1979 that includes age, gender, histologic type, extrathyroidal invasion, and distant metastasis based on a study of 507 patients (3). The AGES (age, gender, extent, size) system from 1987 was based 859 patients at Mayo Clinic between 1946 and 1970 (4). The AMES (age, metastases, extent, and size) system came 1 year later from a Lahey Clinic study involving 821 patients which improved on AGES by including gender (5). As there was no consensus histologic grading scheme when these prognostic systems were created, the Mayo Clinic reanalyzed data from 1779 patients from 1940 to 1989 and developed the MACIS (metastases, age, completeness of resection, invasion, size) system, which excludes histologic grade and includes completeness of primary resection as an independently significant parameter (6).

Although the above systems showed no difference in prediction superiority (7), they are all based on single institution experiences and subject to the limitations therein. As more factors, gene mutations, and clinical features are increasingly utilized in medical decision making, the volume of data in tumor registries is exploding. The fidelity of legacy systems, including those from AJCC that are based on expert panels is called into question as the complexity of analysis necessary to make meaningful interpretations will soon be too cumbersome for humans alone. Can we use machine learning to systematically consider any variable, present and future, to find groups of cancer cases with a similar outcome?

In this study we describe a novel approach using the Ensemble Algorithm for Clustering Cancer Data (EACCD) (8–13) to create prognostic systems for well-differentiated thyroid cancer. We demonstrate the approach by creating a prognostic system on primary tumor, regional lymph nodes, status of distant metastasis, and age using the disease-specific survival data from the Surveillance, Epidemiology, and End Results (SEER) Program of the National Cancer Institute. This system was compared with the widely accepted AJCC staging system for thyroid cancer. We also assess the effect of histologic type and the optimal cutoff for age in producing prognostic systems.

## Materials and Methods

### Data

The SEER database is supported by the National Cancer Institute and collects case data from population-based cancer registries covering approximately 34.6 percent of the U.S. population (14). It contains de-identified data on patient demographics, primary tumor site, tumor morphology, stage at diagnosis, and follow up vital status. For this study we used data of well-differentiated thyroid cancer diagnosed 2004–2010 that were obtained from the November 2017 submission of SEER.

Cases of papillary and follicular thyroid cancer were selected from the SEER 18 databases using the restrictions {ICD-O-3/WHO 2008 = Thyroid} and {Histologic Type ICD-O-3 = 8050, 8260, 8340–8344, 8350, 8450–8460 (for papillary cancer), or Histologic Type ICD-O-3 = 8290, 8330–8335 (for follicular cancer)}. We placed Hurthle cell carcinoma (ICD-O-3 = 8290) into the category of follicular carcinomas, as used in Lim et al. (15). We excluded medullary carcinoma as it is staged differently. Clinically it is often part of genetic syndromes and patients' survival is confounded by its aggressive course. Anaplastic carcinoma is not well-differentiated and uniformly fatal and thus excluded. Furthermore, these 3 types are exceedingly rare and a population-based study on them would be of low utility.

Three datasets were used in this report. Dataset 1, containing 4 prognostic factors, was used to create a prognostic system for thyroid cancer. This system was then compared with the staging system of AJCC. Dataset 2, involving 5 factors, was used to assess the effect of histologic type in creating prognostic systems. Dataset 3, involving 4 factors, was used to explore the optimal cutoff point for age in the prognostic system. Selection of cases, data management, and specifics about factors for each dataset are described as follows.

*Dataset 1* SEER started to record derived AJCC T, N, M according to the 6th AJCC Staging Manual in 2004 (16). The AJCC staging systems of thyroid cancer (17–19) contained further stratified categories (T4a and T4b for T and N1a and N1b for N) that were not available in SEER until 2004. Therefore, dataset 1 contained only cases with diagnosis years from 2004 to 2010 to include these categories for catching up with the latest updates in AJCC, and to ensure a 5-year follow-up through 2015 which was the most recent year before which all case-level data were available in SEER. SEER cause-specific death classification variable (20) was used to capture deaths related to thyroid cancer. Survival time was measured by months. The factors in dataset 1 included tumor size (T), regional lymph nodes (N), status of distant metastasis (M), and age (A). The definition of T, N, and M was from *Adjusted AJCC 6th ed. T, N, M, and Stage* in SEER (16). Age in dataset 1 was treated as a binary variable and contained two categories: A1 (0–54), and A2 (55+). The detailed definition of categories/levels of each factor in dataset 1 was provided in Table 1. We excluded patients with a missing or unknown value on any of the following variables: T, N, M, A, survival time, and SEER cause-specific death classification variable. Specifically, we discarded 26 patients with “T4NOS,” 2894 patients with unknown values of T, 2110 patients with “N1NOS,” 1982 patients with unknown values of N, 1,840 patients with unknown values of M, 4 patents with unknown age, 226 patients with unknown survival time, 131 patients with “Dead (missing/unknown COD)” and 8011 patients with “N/A not first tumor.” We note that patients with an unknown value of one variable are more likely to have unknown values on several other variables.

**Table 1:** Definitions of levels of T, N, M, and A for well-differentiated thyroid cancer patients diagnosed 2004–2010.

**Factors**	**Levels**	**Definitions**
Primary tumor	T0	No evidence of primary tumor
	T1	Tumor 2 cm or less in greatest dimension limited to the thyroid
	T2	Tumor more than 2 cm but not more than 4 cm in greatest dimension limited to the thyroid
	T3	Tumor more than 4 cm in greatest dimension limited to the thyroid or any tumor with minimal extrathyroid extension (e.g., extension to sternothyroid muscle or perithyroid soft tissues)
	T4a	Tumor of any size extending beyond the thyroid capsule to invade subcutaneous soft tissues, larynx, trachea, esophagus, or recurrent laryngeal nerve
	T4b	Tumor invades prevertebral fascia or encases carotid artery or mediastinal vessels
Regional nodes positive	N0	No regional lymph node metastasis
	N1a	Metastasis to Level VI (pretracheal, paratracheal, and prelaryngeal/Delphian lymph nodes)
	N1b	Metastasis to unilateral, bilateral, or contralateral cervical or superior mediastinal lymph nodes
Metastasis	M0	No distant metastasis
	M1	Distant metastasis
Age	A1	0 ≤ Age < 55
	A2	55 ≤ Age

*Refer to SEER Research Data Record Description (21) for specifics of the 3rd column*.

In creating prognostic systems based on dataset 1, our approach applied to combinations instead of individual patients. A combination of prognostic factors is a subset of the data that corresponds to one level of each selected factor. A combination describes certain characteristics of its patients. For example, T1 and N0 produce a combination, denoted by T1N0, which represents a subset of patients whose tumor size is T1 and lymph nodes positive is N0. As in T1N0, we use the notations of levels of factors to denote combinations in this report.

To optimize robustness of statistical techniques, we only kept combinations (in terms of T, N, M, A) each containing at least 25 patients in dataset 1. This left out 33 “rare” combinations (321 cases). Note that 68 cases had T0 and they were excluded since all combinations involving T0 contained fewer than 25 patients. The final dataset 1 consisted of 39 combinations (51,291 cases with a median follow up 90 months).

*Dataset 2* Dataset 2 was derived from dataset 1 by treating the histology (H) as an additional prognostic factor. Two levels were used for histologic type: H1 (follicular) and H2 (papillary). To optimize robustness of statistical techniques, we only kept combinations (in terms of T, N, M, A, H) each containing at least 25 patients. This left out 268 cases from dataset 1. The final dataset 2 consisted of 44 combinations (51,023 cases with a median follow up 90 months). This is the largest dataset that contains combinations (in terms of T, N, M, A, and H) each containing at least 25 patients with diagnosis years from 2004 to 2010.

*Dataset 3* Dataset 3 was also derived from dataset 1 due to consideration of three cutoffs of age 45, 55, and 65. Both 45 and 55 have been used in recent editions of AJCC, and 65 was considered because of its general use in the literature for stratifying young and old patients. We required that each combination from T, N, M, and any cutoff contain at least 25 patients. This left out 186 cases from dataset 1. The final dataset contains 51,105 cases (with a median follow up 90 months), which is the largest dataset that contains combinations (in terms of T, N, M, A with any of the three cutoffs) each containing at least 25 patients with diagnosis years from 2004 to 2010.

### EACCD

The EACCD is a machine learning algorithm designed to partition survival data. It consists of 3 main steps. (1) Defining initial dissimilarities: This step defines the initial dissimilarity between survival functions of any two combinations. (2) Obtaining learned dissimilarities: This step uses initial dissimilarities and an ensemble learning process to obtain learned dissimilarities between combinations, which are more data-driven than the initial dissimilarities. (3) Applying hierarchical clustering analysis: This step clusters the combinations by the learned dissimilarities and a linkage method.

There are several approaches for each step. In this paper, the initial dissimilarity between two combinations is defined by an effect size based on Gehan-Wilcoxon test statistic (22); the ensemble learning process is based on the two-phase Partitioning Around Medoids algorithm (23); and the complete linkage method (24) is chosen for hierarchical clustering. These and a detailed description of EACCD are given in the Supplementary Material.

### Prognostic Systems

Dendrograms can be cut horizontally to generate prognostic groups that serve the same role as the staging groups in the TNM. We cut dendrograms according to the C-index (25), which estimates the probability that a subject who experienced an event (e.g., death) in an earlier time had a shorter predicted time than a subject who experienced the event in a later time. A higher C-index implies a higher accuracy in survival prediction. In general, the curve of the C-index vs. the number of groups increases for relatively small numbers of groups and then quickly plateaus as more groups are generated. The C-index curve can be used to find the optimal number of groups (denoted by n^*^) for the model, which is around the “knee” point of the curve and balances the simplicity and the accuracy of the system. The C-index value at n^*^ is usually close to the maximum C-index that corresponds to the maximum number of groups, i.e., the number of combinations based on the selected factors. Survival curves using the Kaplan-Meier estimates (26) are plotted for the prognostic groups to visually evaluate the survival difference among the prognostic groups. The survival curves for n^*^ groups should not overlap. The final prognostic system is a collection of the dendrogram, the group assignment, the C-index, and the survival curves for the prognostic groups. In this report a prognostic system is sometimes denoted by notations of the factors involved in the system.

## Results

### Prognostic System on the Basis of T, N, M, A

**Figure 1:**
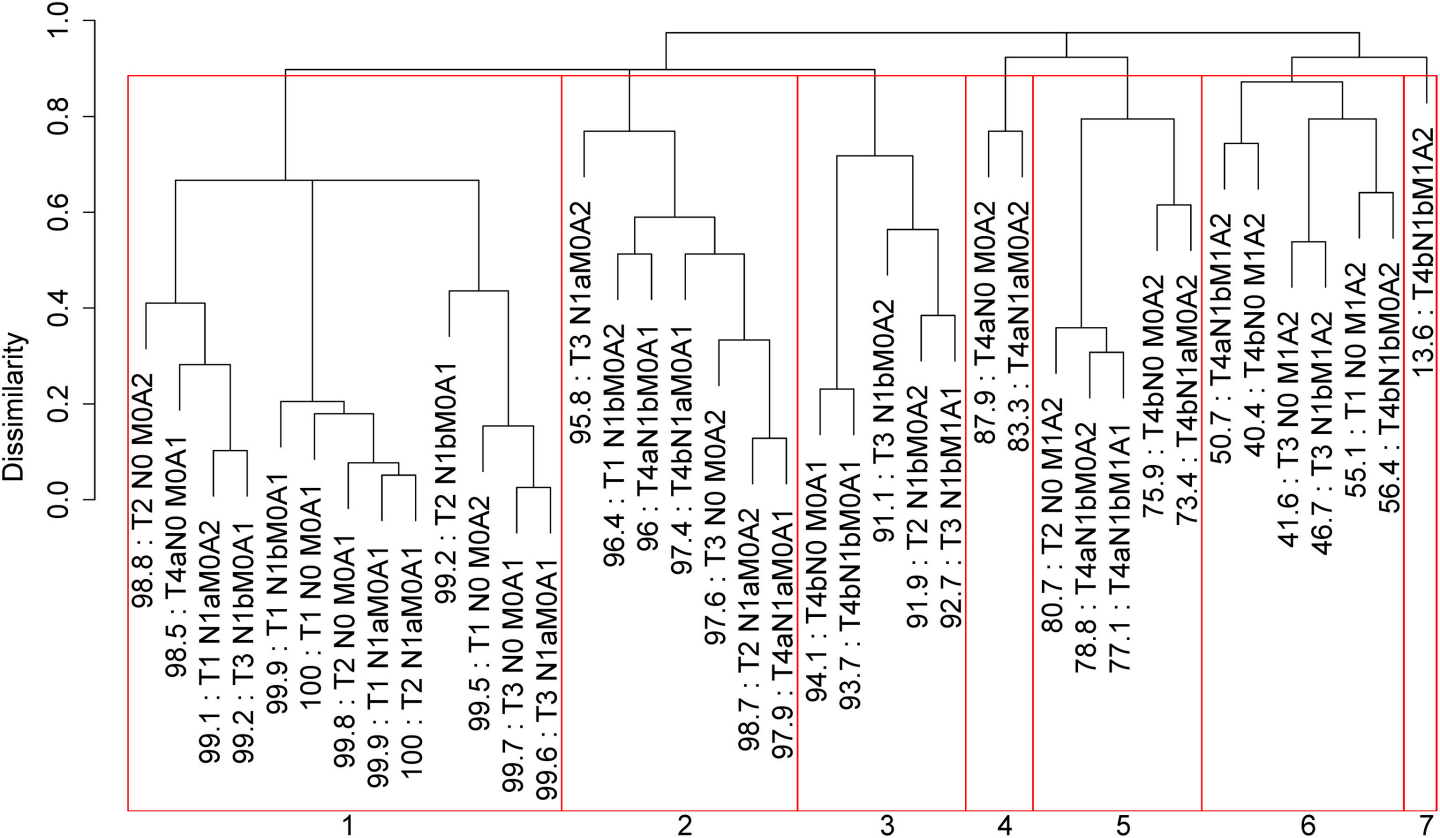
Dendrogram for dataset 1 and cutting the dendrogram according to C-index. Running EACCD results in the tree-structured dendrogram, shown in the black color in the figure. A 5-year disease-specific survival rate in percentage is given beneath each combination. Cutting the dendrogram according to n^*^ = 7 in Figure 2 creates 7 prognostic groups, shown in red square boxes. Listed on the bottom are the group numbers.

**Figure 2:**
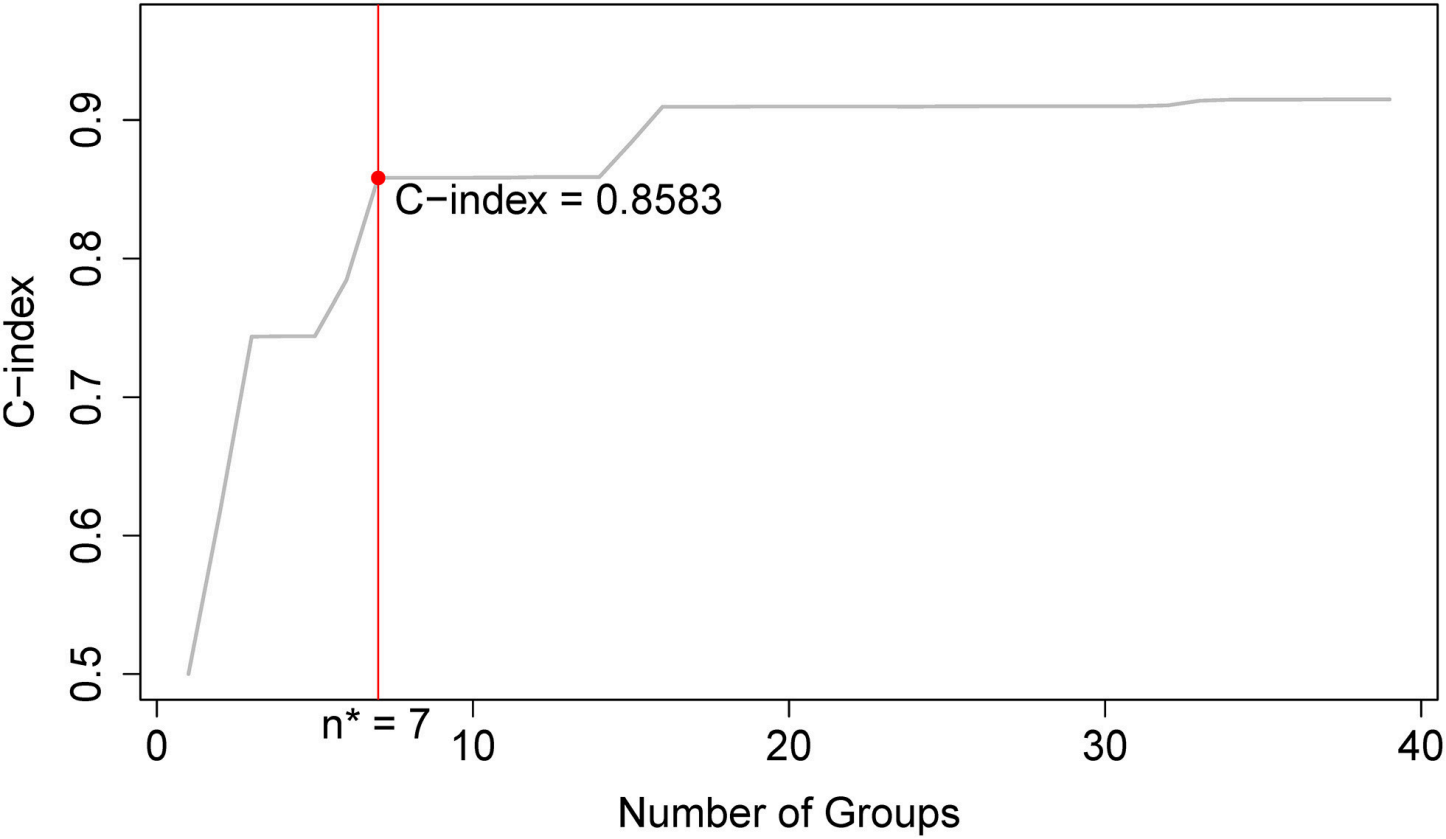
C-index curve based on the dendrogram in Figure 1. The highest knee point of the curve corresponds to 16 groups and the C-index value of around 0.9. However, 16 groups of patients obtained from cutting the dendrogram have overlapping survival curves and thus should not be used. Therefore, the optimal number n^*^ is smaller than 16. Fifteen groups also yield overlapping survival curves and should not be used either. The next knee point is at 7 groups with well-separated survival curves and a C-index of 0.8583. Thus, the optimal n^*^ = 7.

**Figure 3:**
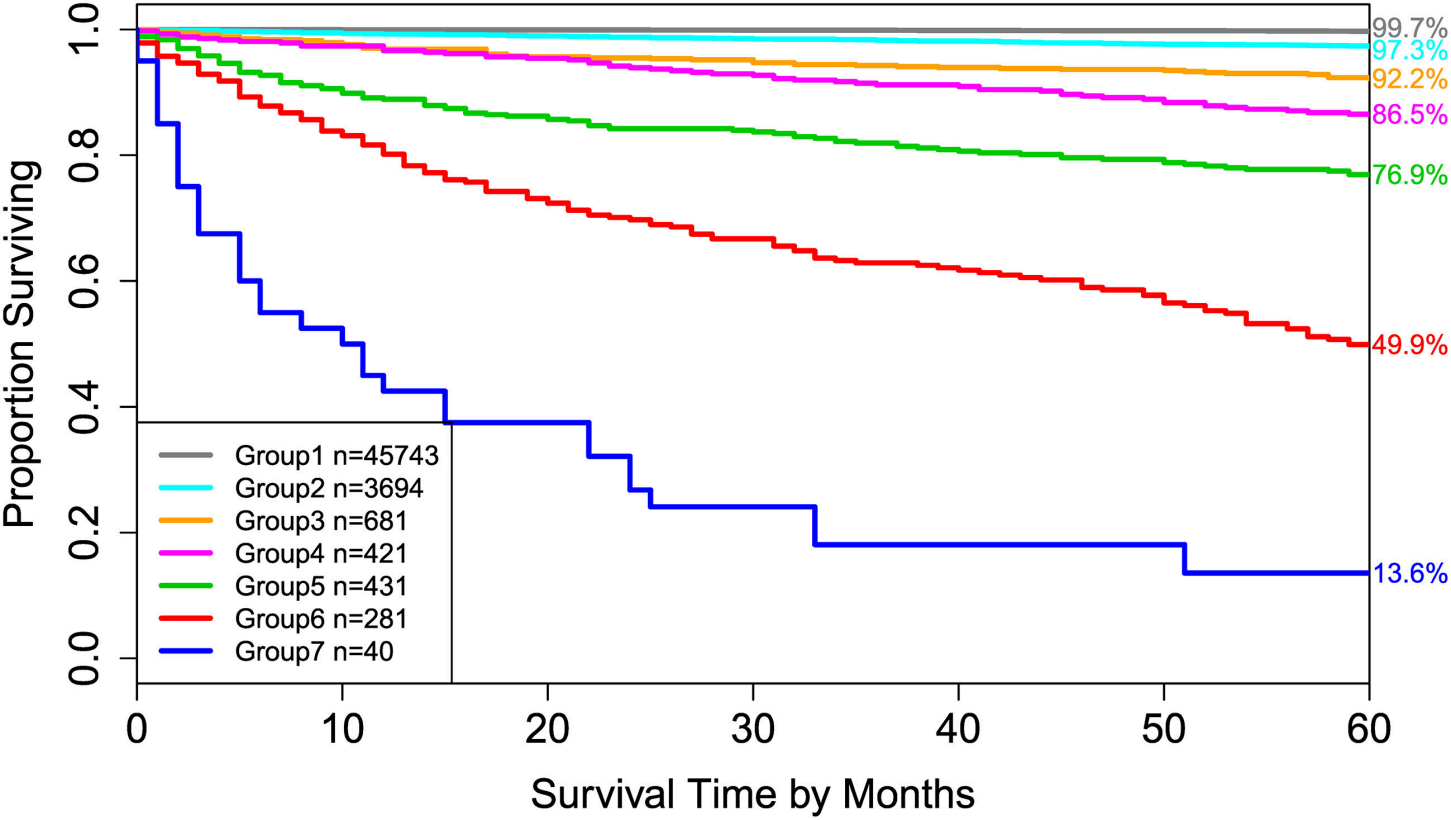
Disease-specific survival of 7 prognostic groups in Figure 1. The 5-year disease-specific survival rates for 7 groups are listed on the right side of the figure.

Applying EACCD to dataset 1 yields the dendrogram in Figure 1. The C-index curve (C-index vs. number of groups) is shown in Figure 2 and can be used to find the optimal number of prognostic groups n^*^. The highest knee point of the curve corresponds to 16 groups and a C-index value of around 0.9. However, 16 groups obtained from cutting the dendrogram have overlapping survival curves and thus should not be used. Then n^*^ is smaller than 16, since survival curves of more than 16 groups will also overlap. Fifteen groups yield overlapping survival curves and should not be used either. The next knee point is at 7 groups with non-overlapping survival curves and a C-index of 0.8583 and thus we choose n^*^ = 7. Cutting the dendrogram into 7 groups is shown in Figure 1 and the survival curves of the 7 groups are plotted in Figure 3. Clearly, group 1, group 2, …, group 7 have well-separated curves and have a decreasing survival as the group number increases. For convenience, the detailed definition for all 7 groups is restated in the 5th column of Table 2.

**Table 2:** EACCD and AJCC stratification of dataset 1 containing the SEER well-differentiated (papillary + follicular) thyroid cancer patients diagnosed 2004–2010.

**T**	**N**	**M**	**A**	**EACCD prognostic group**	**8th AJCC Staging Manual staging group**
T1	N0	M0	A1	1	I
T1	N0	M0	A2	1	I
T1	N1a	M0	A1	1	I
T1	N1a	M0	A2	1	II
T1	N1b	M0	A1	1	I
T2	N0	M0	A1	1	I
T2	N0	M0	A2	1	I
T2	N1a	M0	A1	1	I
T2	N1b	M0	A1	1	I
T3	N0	M0	A1	1	I
T3	N1a	M0	A1	1	I
T3	N1b	M0	A1	1	I
T4a	N0	M0	A1	1	I
T1	N1b	M0	A2	2	II
T2	N1a	M0	A2	2	II
T3	N0	M0	A2	2	II
T3	N1a	M0	A2	2	II
T4a	N1a	M0	A1	2	I
T4a	N1b	M0	A1	2	I
T4b	N1a	M0	A1	2	I
T2	N1b	M0	A2	3	II
T3	N1b	M0	A2	3	II
T3	N1b	M1	A1	3	II
T4b	N0	M0	A1	3	I
T4b	N1b	M0	A1	3	I
T4a	N0	M0	A2	4	III
T4a	N1a	M0	A2	4	III
T2	N0	M1	A2	5	IVB
T4a	N1b	M0	A2	5	III
T4a	N1b	M1	A1	5	II
T4b	N0	M0	A2	5	IVA
T4b	N1a	M0	A2	5	IVA
T1	N0	M1	A2	6	IVB
T3	N0	M1	A2	6	IVB
T3	N1b	M1	A2	6	IVB
T4a	N1b	M1	A2	6	IVB
T4b	N0	M1	A2	6	IVB
T4b	N1b	M0	A2	6	IVA
T4b	N1b	M1	A2	7	IVB

Roughly, it is seen that 5 year disease-specific survival rates within each of the 7 red boxes in Figure 1 are close to each other and they differ between any two red boxes. Therefore, the red boxes in Figure 1 provide a good grouping of the patients. Figure 3 shows well-separated survival curves of 7 groups from the 7 red boxes.

The dendrogram with cutting in Figure 1, the groups in Table 2, and the survival curves in Figure 3 define one prognostic system for well-differentiated thyroid cancer that incorporates T, N, M, and A. For simplicity, this system is denoted by TNMA. We call each of the 7 groups a prognostic group.

**Figure 4:**
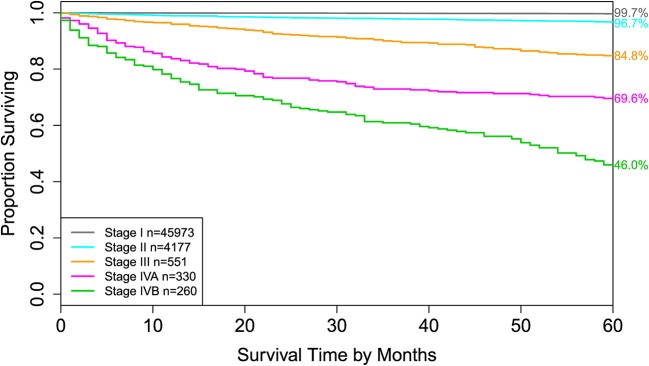
Disease-specific survival of AJCC stage groups defined in the 6th column in Table 2. The 5-year disease-specific survival rates for 5 stages are listed on the right side of the figure.

For comparison, the 8th edition of AJCC divides dataset 1 into 5 groups (Figure 4 and the sixth column of Table 2). Calculation shows that the C-index of staging system TNMA from AJCC 8th edition is 0.8387. The *p*-value of the C-index based test (27) for testing the difference between the prediction accuracy of the EACCD prognostic system TNMA (7 groups, C-index = 0.8583) and the AJCC 8th edition staging system TNMA (5 groups, C-index 0.8387) is 7.8 × 10^−7^. This shows that on the basis of dataset 1, the EACCD generated prognostic system TNMA has a significantly higher prediction accuracy than the staging system of AJCC 8th edition.

### Effect of Histology

**Figure 5:**
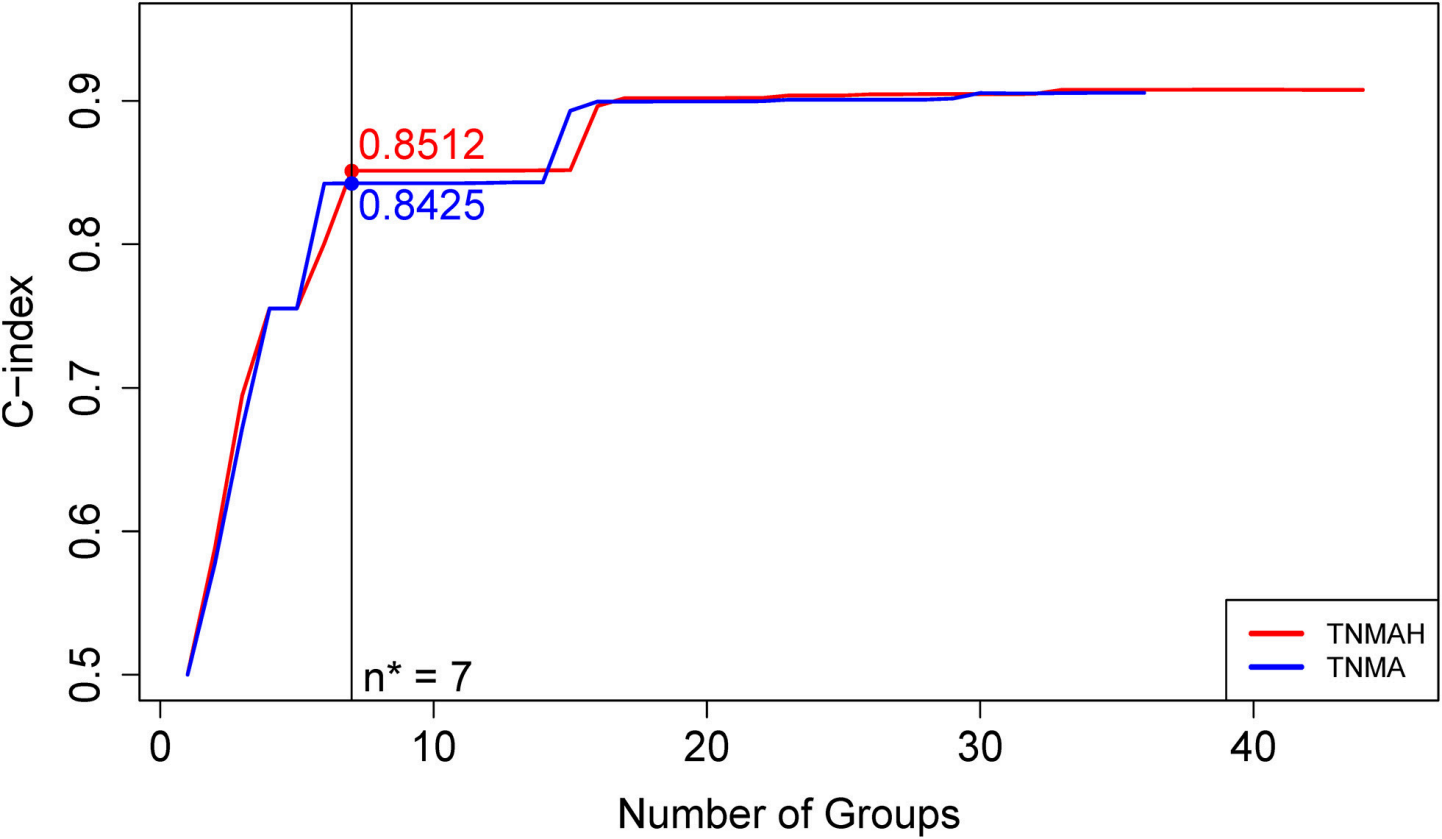
C-index curves based on dataset 2. The red curve is the C-index curve from the dendrogram using 5 factors T, N, M, A, and H. The blue curve is the C-index curve from the dendrogram using 4 factors T, N, M, and A. The optimal number of groups is 7 for both systems, and the corresponding C-index values for the red and blue curves are, respectively, 0.8512 and 0.8425 (*p*-value = 0.00052).

To explore the effect of histology on prognostic systems, we used EACCD to build the prognostic systems on the basis of dataset 2 for the following two sets of factors: {T, N, M, A} and {T, N, M, A, H}. (Dataset 2 meets the requirement of at least 25 patients in each combination for each set, while dataset 1 does not. Therefore, dataset 2 can be used for a fair assessment of these two sets of factors on the same data.) Figure 5 plots C-index curves, based on dataset 2, for the two scenarios. The optimal number of groups is 7 for both sets, and the corresponding C-index for {T, N, M, A} and {T, N, M, A, H} is 0.8425 and 0.8512, respectively. The *p*-value of the C-index based test (27) for testing the difference in prediction of the two prognostic systems is 0.00052, showing that inclusion of histology significantly improves prediction accuracy compared to the system based on T, N, M, A alone.

### Cutoff Point of Age

In the above, we have used 55 as a cutoff for age. With different cutoff values of age, prognostic systems can be created and compared in terms of prediction accuracies. We provide such an analysis for cutoffs 45, 55, and 65 on the basis of dataset 3, which is the largest dataset that allows at least 25 patients in any considered combination. From our approach in creating prognostic systems, the cutoffs 45, 55, and 65 lead to three prognostic systems that have, respectively, a C-index 0.7710, 0.8394, and 0.8047. The *p*-value of the test in Kang et al. (27) for testing the difference in prediction between the system with cutoff 55 and the system with cutoff 45 and 65 is 5.4 × 10^−5^ and 3.3 × 10^−10^, respectively. The prognostic system based on age cutoff 55 performs significantly better than the systems with cutoff 45 or 65. Therefore, adjusting for T, N, and M, 55 is a good cutoff if dichotomizing age is required (i.e., for clinical reasons).

Note that all three systems have 6 prognostic groups, rather than 7 groups as in the system TNMA based on dataset 1. This difference is due to the fact that dataset 3 is a subset of dataset 1 and those patients in dataset 1 but not in dataset 3 are from small combinations which are mainly assigned to high-risk groups (groups 5, 6, and 7) in the 5th column in Table 2. For example, 33 T1N0M1A2 cases, 31 T3N1bM1A2 cases, and 32 T4bN0M1A2 cases, all from group 6, are excluded from dataset 3. These patients have a high death rate and excluding them can affect the group assignment and predictive accuracy of the systems based on dataset 3.

## Discussion

### Histology

We showed that when histology was added into the pool of T, N, M, and A, the prediction accuracy increased (*p*-value = 0.00052). However, the improvement was not dramatic, as shown by their small difference in C-index values in Figure 5. This is consistent with earlier findings (28, 29).

We now examine how the EACCD system TNMA classifies the patients in terms of their status of histology. Table 3 describes how patients with different types of histology and different AJCC stages are distributed across the prognostic groups of the EACCD system TNMA. For each type (papillary and follicular), the upper right and lower left corners of the table are filled with 0. Low and high stages correspond to low and high-risk prognostic groups, respectively. Therefore, the EACCD grouping and AJCC staging are strongly positively correlated.

**Table 3:** Contingency table between EACCD grouping and AJCC staging on the basis of T, N, M, A, and dataset 1 that contains the SEER well-differentiated (papillary + follicular) thyroid cancer patients diagnosed 2004–2010.

**AJCC/EACCD**	**1**	**2**	**3**	**4**	**5**	**6**	**7**	**Total**
**FOLLICULAR**								
I	3,668	3	25	0	0	0	0	3,696
II	5	659	15	0	2	0	0	681
III	0	0	0	33	2	0	0	35
IVA	0	0	0	0	42	7	0	49
IVB	0	0	0	0	16	67	13	96
Total	3,673	662	40	33	62	74	13	4,557
**PAPILLARY**								
I	41,634	463	180	0	0	0	0	42,277
II	436	2,569	461	0	30	0	0	3,496
III	0	0	0	388	128	0	0	516
IVA	0	0	0	0	192	89	0	281
IVB	0	0	0	0	19	118	27	164
Total	42,070	3,032	641	388	369	207	27	46,734

### Cutoff of Age

It is known that age is a very important factor that has a strong correlation with the outcome of differentiated thyroid cancer patients, i.e., increasing patient age is significantly associated with increasing mortality (after adjustment for certain characteristics) (28–30). Since its second edition, the AJCC staging system for thyroid cancer has been including age in addition to T, N, and M. With AJCC, age has been treated as a dichotomous variable, with a cutoff at 45 for earlier AJCC editions including the 7th and 55 for the current 8th edition.

The concept of cutoffs of age has been challenged recently in a large number of studies that use statistical modeling techniques to make inconsistent conclusions (30). In particular, in a study of 31,802 patients with papillary thyroid cancer, Adam et al. used the Cox proportional hazards modeling with restricted cubic splines to show that age was significantly associated with survival, without an apparent cutoff. When applied to survival data, statistical modeling methods (e.g., Cox regression models) usually focus on optimal fitting to the data. And correspondingly, they favor continuous variables (if variables can be treated as continuous) instead of discretizing continuous variables, which can cause a loss in the information contained in the data.

On the other hand, when stratifying the data is needed, such as staging the patients as AJCC and grouping patients by EACCD as shown in this research, obtaining a well-defined rule to classify the patients into various categories becomes another important issue, in addition to achieving a high survival prediction accuracy. In this scenario where both stratification and prediction are of main concern, treating age as continuous is inconvenient. The simplest approach is then to find a cutoff for age. In this study, we showed that when using EACCD to groups, with a high prediction accuracy, 51,291 patients of well-differentiated thyroid cancer, 55 is the best cutoff of age among the three choices of 45, 55, and 65. This finding supports the use of 55 as an age cutoff in the current AJCC staging system (8th edition) for thyroid cancer.

### Comparing EACCD vs. AJCC 8th Edition

The EACCD prognostic system TNMA based on dataset 1 can be compared with the AJCC staging system in terms of stratification and prediction. We showed earlier that the EACCD system TNMA (C-index = 0.8583) has a significantly higher survival prediction accuracy than the AJCC staging system (C-index = 0.8387). Below we compare the two systems by examining how they stratify patients.

There is a strong inter-system association between AJCC staging and EACCD grouping. Table 4 presents the distribution of patients of each AJCC stage over the 7 groups of EACCD system TNMA. Note that the upper right and lower right corners of the table are filled with 0. We see that the higher stage the patient is assigned to by the AJCC system, the higher-risk group the patient is assigned to by the EACCD, and vice versa. In fact, the assignment to ordered stages and the assignment to ordered groups have a Spearman's rank correlation of 0.8798 with a *p*-value of 1.7 × 10^−13^.

**Table 4:** Contingency table between EACCD grouping and AJCC staging on the basis of T, N, M, A, and dataset 1 that contains the SEER well-differentiated (papillary + follicular) thyroid cancer patients diagnosed 2004–2010.

**AJCC/EACCD**	**1**	**2**	**3**	**4**	**5**	**6**	**7**	**Total**
**DIFFERENTIATED (FOLLICULAR** **+PAPILLARY)**								
I	45,302	466	205	0	0	0	0	45,973
II	441	3,228	476	0	32	0	0	4,177
III	0	0	0	421	130	0	0	551
IVA	0	0	0	0	234	96	0	330
IVB	0	0	0	0	35	185	40	260
Total	45,743	3,694	681	421	431	281	40	51,291

EACCD has a more rigorous grouping scheme than AJCC. A notable combination is T4aN1bM1A1 (32 cases), which is considered Stage II according to the AJCC system. This combination has a 5-year disease-specific survival rate of 77.1%, yet the Stage II 5-year disease-specific survival rate is more than 95% (based on dataset 1). In contrast, EACCD assigns T4aN1bM1A1 to group 5 of the system TNMA whose 5-year disease-specific survival rate is around 76% (based on dataset 1).

In the EACCD system TNMA, a combination with a lower level of one selected factor (adjusting for the other three factors) is almost always assigned into a prognostic group with an equal or more favorable survival. The only two exceptions are: T1N0M1A2 (group 6, 33 cases, 5-year survival = 55.1%) and T2N0M1A2 (group 5, 35 cases, 5-year survival = 80.7%), and T4bN0M0A1 (group 3, 121 cases, 5-year survival = 94.1%) and T4bN1aM0A1 (group 2, 83 cases, 5-year survival = 97.4%). Though the assignments are concordant with the observed survival, they may counter to our usual understanding of levels of factors. This problem is caused by inaccurate estimates of survival due to small sample sizes and will be corrected when more data are available. We note that this problem does not occur with AJCC staging.

In summary, the EACCD prognostic system TNMA has a higher prognostic accuracy than the AJCC staging system; in stratification of patients, EACCD grouping and AJCC staging are positively associated though each has its own advantages and disadvantages.

### Comparing EACCD With Other Models

Other efforts have been made to expand the AJCC staging system by integrating additional factors. Two major approaches are available in the literature, one based on Cox regression modeling (28) and the other on tree modeling (29).

Cox regression modeling, focuses on optimal fitting to the data, can achieve a high accuracy in survival prediction. The main downside is that no clear rule can be extracted from the output (e.g., the nomogram) to stratify patients into risk groups analogous to AJCC stages.

Traditional survival tree modeling, partitioning the space of values of factors into disjoint and non-overlapping regions, can be used to explicitly define prognostic groups. However, tree models in general do not provide a high prediction accuracy.

In contrast, the EACCD approach introduced in this paper computes the survival difference between any two cohorts of patients and utilizes these differences to stratify patients, where the number of groups from stratification is determined by potentially largest C-index. Therefore, this approach takes into account both stratification and prediction.

### Limitations

The gold standard disease-specific survival data were used in this study. Although the SEER cause-specific death classification is determined by also taking into account other elements (e.g., tumor sequence, site of the original cancer diagnosis, and comorbidities), death certificate errors can be problematic in estimating the cause-specific survival. Another limitation is including combinations with at least 25 cases, which excludes some combinations that contain a few patients with very poor prognosis. The impact of this requirement on combination sizes will be minimized as more data become available.

## Conclusions

We have introduced a novel machine learning approach EACCD to create prognostic systems for cancer patients. Using data from the SEER national cancer registry and machine learning methods, we were able to create a computational prognostic system TNMA based on T, N, M, and age for differentiated thyroid carcinoma that had a significantly higher survival prediction accuracy than the AJCC staging scheme. We showed that adding histology into the model TNMA only improved performance slightly. We also showed that 55 is the best cutoff of age in our approach of stratifying patients. Although the EACCD prognostic system TNMA has a significantly higher prediction accuracy in survival, the EACCD grouping and AJCC staging are strongly positively correlated.

The EACCD approach can be applied to validate/expand other prognostic systems utilized in clinical practice such as EORTC, AGES, AMES, and MACIS. Future interesting applications of EACCD could include studying molecular markers, such as T1799A point BRAF mutation, TERT mutation, and HRAS mutation (31) as these become available in cancer registries. As new variables/factors become important for clinical decisions, they can be integrated with others in a data-driven approach by EACCD to provide timely refinements and further optimization in patient classification and outcome prediction.

## Author Contributions

CY and DC designed the study. HW collected the data. HW and DC analyzed the data and prepared the tables and figures. CY, LG, HW, and DC prepared the manuscript. CY, LG, and MH reviewed clinical implications of the results. All authors reviewed the manuscript.

### Conflict of Interest Statement

The authors declare that the research was conducted in the absence of any commercial or financial relationships that could be construed as a potential conflict of interest.
