# Supplementary material for: Creating Prognostic Systems for Well-Differentiated Thyroid Cancer Using Machine Learning
Source: Front Endocrinol (Lausanne). 2019 May 8;10:288. doi: 10.3389/fendo.2019.00288 (PMC6517862; doi:10.3389/fendo.2019.00288)
Supplement: Supplementary file 1 [file Data_Sheet_1.pdf]

Appendix

EACCD

The EACCD (Chen 2009) is an unsupervised learning algorithm designed to partition patients who have records on the survival time and censoring status as well as measurements on a sequence of selected categorical variables. Development has targeted on its application and improvement (Qi *et al*. 2013, Qi *et al*. 2014, Chen *et al*. 2016, Wang H *et al*. 2017, Hueman *et al*. 2018, Wang et al. 2018). The algorithm consists of 3 steps: defining initial dissimilarities (in terms of the difference between survival functions) between combinations, computing learned dissimilarities, and performing hierarchical clustering of the combinations. One convenient version of the algorithm is seen as follows:

Given a collection of combinations $\{Comb_1, Comb_2, \ldots\ldots, Comb_n\}$ and nonnegative weights $w_1, w_2, \ldots, w_n$ with $\sum_{k=1}^{n} w_k = 1$.

1. Define the initial dissimilarity $dis_0(com_j, com_j)$ for any pair com_i and com_j.
2. For each k with $1 \leq k \leq n$, apply the two-phase PAM and the initial dissimilarities in Step 1 to partition combinations into k clusters, and define $\delta_k(i,j) = 1$ if com_i and com_j are not assigned into the same cluster and $\delta_k(i,j) = 0$ otherwise. Define the learned dissimilarity $dis(Comb_i, Comb_j) = \sum_{k=1}^{n} w_k \delta_k(i,j)$.
3. Perform hierarchical clustering to cluster the combinations.

In Step 1, the initial dissimilarity can be defined as the value of a test statistic, such as the log-rank test statistic, Gehan-Wilcoxon test statistic, and Tarone-Ware test statistic. When the sizes of combinations are big, better initial dissimilarities can be defined by effect-size based measures, such as hazard ratios and Mann-Whitney parameters (Wang 2017, 2018).

Step 2 utilizes initial dissimilarities in Step 1 and an ensemble process to compute the learned dissimilarities, which are more data driven than the initial dissimilarities. The two-phase Partitioning Around Medoids algorithm (PAM) (Kaufman & Rousseeuw 1990) is used in the ensemble process to partition combinations. The results from PAM are then combined to produce the learned dissimilarity, which is simply the weighted percentage of the times two combinations are not placed into the same cluster by the PAM algorithm. One simple selections of weights is $w_k = 1/kw$ with $w = 1/1 + 1/2 + \ldots + 1/n$ for $k = 1, 2, \ldots, n$. In early versions EACCD, learned dissimilarities were obtained by averaging the results from many runs of partition methods, which could take a long time to complete if a huge number of runs were used. In contrast, Step 2 above only requires to run PAM n times, a number determined by the number of combinations.

Step 3 clusters the combinations by the learned dissimilarities from Step 2 and a linkage method. Single linkage, average linkage, complete linkage, minimax linkage (Hastie 2013, Bien 2011), or other agglomerative hierarchical clustering methods may be used in this step. The primary output is a dendrogram that provides a graphical summary of patients' survival based on the levels of prognostic factors or variables.

In this report, the initial dissimilarity in Step 1 is based on the "Gehan effect size" described below; the weights in Step 2 are chosen to be $w_1 = \ldots = w_K = 1/n$; and the complete linkage method is used in Step 3.

Gehan Effect Size

Gehan-Wilcoxon test (Gehan 1965) is one of the most popular tests in detecting the difference between two populations' survival. Gehan-Wilcoxon test statistic can be represented as a weighted difference in the estimated hazards (Fleming & Harrington 1991). Let t_1,..., t_J be the observed death times, then the weighted statistic is

$$U = \sum_{j=1}^{J} \frac{Y_j}{n_1+n_2} (D_{1j} - D_j \frac{Y_{1j}}{Y_j}),$$

where n_1 and n_2 are, respectively, the number of patients who died and at risk at t_j in population i (i= 1, 2), D_{1j} and Y_{1j} are, respectively, the number of patients who died and at risk at t_j in population i (i= 1, 2), and D_j and Y_j are, respectively, the number of patients who died and at risk at t_j in both populations. It can be shown, in probability,

$$\frac{n_1 + n_2}{n_1 n_2} U \rightarrow \int S_1(t)(1 - G_1(t))S_2(t)(1 - G_2(t))(\lambda_1(t) - \lambda_2(t))dt$$

where $S_i(t)$, $G_i(t)$, and $\lambda_i(t)$ are, respectively, the survival function, cumulative distribution function of censoring, and hazard function in population i (i= 1, 2). We call the integral on the right hand side the Gehan effect size, which does not depend on sample sizes. As a result, $\frac{n_1+n_2}{n_1 n_2} U$ estimates the weighted differences in hazards and can be used as a measure of dissimilarity between two populations' survival functions.
